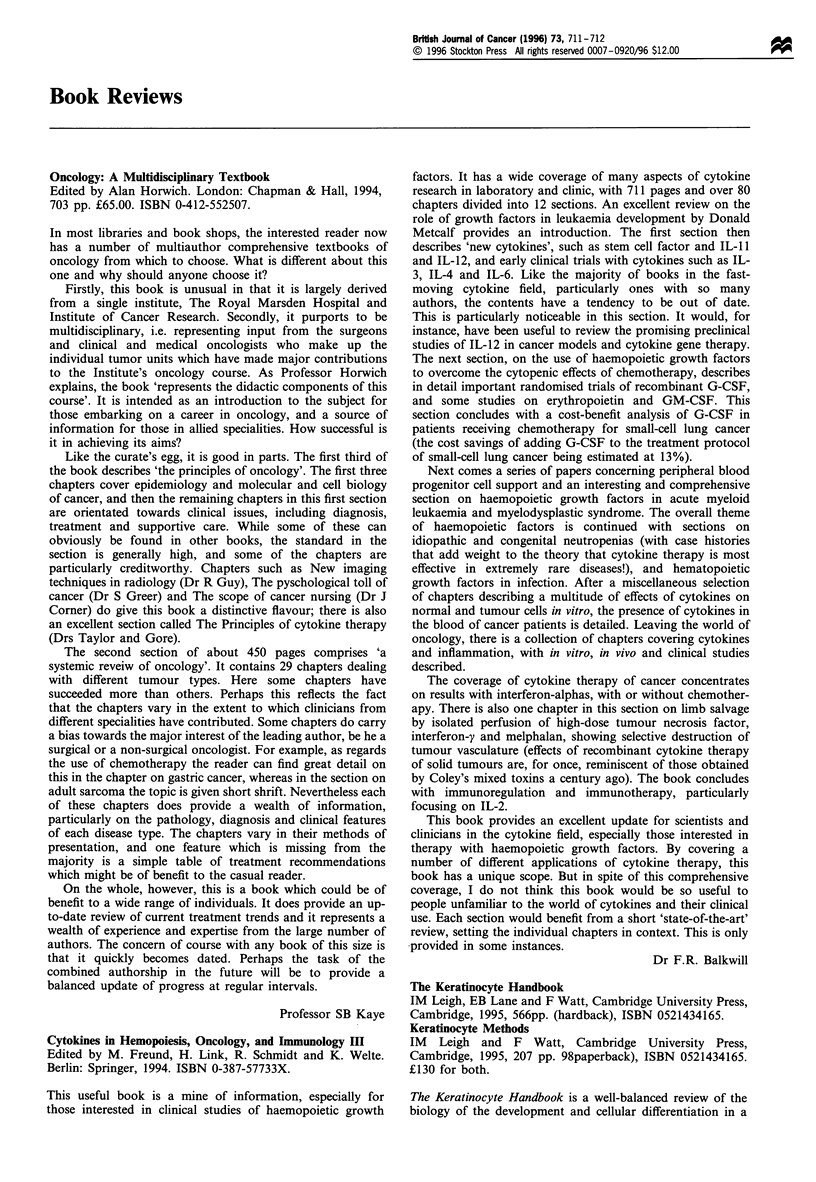# Cytokines in Hemopoiesis, Oncology, and Immunology III

**Published:** 1996-03

**Authors:** F.R. Balkwill


**Cytokines in Hemopoiesis, Oncology, and Immunology III**
Edited by M. Freund, H. Link, R. Schmidt and K. Welte. Berlin: Springer, 1994. ISBN 0-387-57733X.

This useful book is a mine of information, especially for those interested in clinical studies of haemopoietic growth factors. It has a wide coverage of many aspects of cytokine research in laboratory and clinic, with 711 pages and over 80 chapters divided into 12 sections. An excellent review on the role of growth factors in leukaemia development by Donald Metcalf provides an introduction. The first section then describes 'new cytokines', such as stem cell factor and IL-11 and IL-12, and early clinical trials with cytokines such as IL-3, IL-4 and IL-6. Like the majority of books in the fast-moving cytokine field, particularly ones with so many authors, the contents have a tendency to be out of date. This is particularly noticeable in this section. It would, for instance, have been useful to review the promising preclinical studies of IL-12 in cancer models and cytokine gene therapy. The next section, on the use of haemopoietic growth factors to overcome the cytopenic effects of chemotherapy, describes in detail important randomised trials of recombinant G-CSF, and some studies on erythropoietin and GM-CSF. This section concludes with a cost-benefit analysis of G-CSF in patients receiving chemotherapy for small-cell lung cancer (the cost savings of adding G-CSF to the treatment protocol of small-cell lung cancer being estimated at 13%).

Next comes a series of papers concerning peripheral blood progenitor cell support and an interesting and comprehensive section on haemopoietic growth factors in acute myeloid leukaemia and myelodysplastic syndrome. The overall theme of haemopoietic factors is continued with sections on idiopathic and congenital neutropenias (with case histories that add weight to the theory that cytokine therapy is most effective in extremely rare diseases!), and hematopoietic growth factors in infection. After a miscellaneous selection of chapters describing a multitude of effects of cytokines on normal and tumour cells *in vitro*, the presence of cytokines in the blood of cancer patients is detailed. Leaving the world of oncology, there is a collection of chapters covering cytokines and inflammation, with *in vitro*, *in vivo* and clinical studies described.

The coverage of cytokine therapy of cancer concentrates on results with interferon-alphas, with or without chemotherapy. There is also one chapter in this section on limb salvage by isolated perfusion of high-dose tumour necrosis factor, interferon-$\gamma$ and melphalan, showing selective destruction of tumour vasculature (effects of recombinant cytokine therapy of solid tumours are, for once, reminiscent of those obtained by Coley's mixed toxins a century ago). The book concludes with immunoregulation and immunotherapy, particularly focusing on IL-2.

This book provides an excellent update for scientists and clinicians in the cytokine field, especially those interested in therapy with haemopoietic growth factors. By covering a number of different applications of cytokine therapy, this book has a unique scope. But in spite of this comprehensive coverage, I do not think this book would be so useful to people unfamiliar to the world of cytokines and their clinical use. Each section would benefit from a short 'state-of-the-art' review, setting the individual chapters in context. This is only provided in some instances.

Dr F.R. Balkwill